# Strength, Endocrine, and Body Composition Alterations across Four Blocks of Training in an Elite 400 m Sprinter

**DOI:** 10.3390/jfmk6010025

**Published:** 2021-03-09

**Authors:** Amit Batra, Alex B. Wetmore, W. Guy. Hornsby, Patrycja Lipinska, Zbigniew Staniak, Olga Surala, Michael H. Stone

**Affiliations:** 1 Department of Sport, Exercise, Recreation, and Kinesiology, East Tennessee University, Johnson City, TN 36714, USA; STONEM@mail.etsu.edu; 2 Department of Athletics, Westminster College, Salt Lake City, UT 16172, USA; WETMORE@mail.etsu.edu; 3 College of Physical Activity and Sport Sciences, West Virginia University, Morgantown, WV 26505, USA; william.hornsby@mail.wvu.edu; 4 Institute of Physical Education, University of Bydgoszcz, 85-064 Bydgoszcz, Poland; patlip@ukw.edu.pl; 5 Department of Biomechanics, Institute of Sport, National Research Institute, 01-982 Warsaw, Poland; zbigniew.staniak@insp.waw.pl; 6 Department of Nutrition Physiology and Dietetics, Institute of Sport-National Research Institute, 02-776 Warsaw, Poland; olga.surala@insp.waw.pl

**Keywords:** periodization, sprinter, track and field, athlete monitoring, endocrine, force plate

## Abstract

The ability to produce force rapidly has the potential to directly influence sprinting performance through changes in stride length and stride frequency. This ability is commonly referred to as the rate of force development (RFD). For this reason, many elite sprinters follow a combined program consisting of resistance training and sprint training. The purpose of this study was to investigate the strength, endocrine and body composition adaptations that occur during distinct phases of a block periodized training cycle in a 400 m Olympic level sprinter. The athlete is an elite level 400 m male sprinter (age 31 years, body mass: 74 kg, years of training: 15 and Personal Best (PB): 45.65 s). This athlete completed four distinct training phases of a block periodized training program (16 weeks) with five testing sessions consisting of testosterone:cortisol (T/C) profiles, body composition, vertical jump, and maximum strength testing. Large fluctuations in T/C were found following high volume training and the taper. Minor changes in body mass were observed with an abrupt decrease following the taper which coincided with a small increase in fat mass percentage. Jump height (5.7%), concentric impulse (9.4%), eccentric impulse (3.4%) and power ratio (18.7%) all increased substantially from T1 to T5. Relative strength increased 6.04% from T1 to T5. Lastly, our results demonstrate the effectiveness of a competitive taper in increasing physiological markers for performance as well as dynamic performance variables. Block periodization training was effective in raising the physical capabilities of an Olympic level 400 m runner which have been shown to directly transfer to sprinting performance.

## 1. Introduction

The 400 m sprint is a speed endurance event that demands a high level of anaerobic metabolism, buffering capacity and aerobic processes to maintain maximum velocity [1,2,3,4]. Although stride frequency and stride length have been shown to influence sprinting speed, stride length seems to be the more important biomechanical parameter when distinguishing between levels of performance in 400 m races [5]. Elite sprinters have demonstrated the ability to apply greater forces into the ground, resulting in longer stride lengths, faster stride frequencies, and subsequently, faster sprint times compared to less experienced sprinters [6,7]. Although the 400 m race is classified as “sprint distance” it is characterized by unique metabolic, neuromuscular, and technical requirements in comparison to 100 and 200 m races [5,8].

Agreement exists that, from a bioenergetics/metabolic standpoint, anaerobic capacity is the main factor discriminating 400 m performance [2]. Nevertheless, the need to generate high forces in a small amount of time underscores the importance of qualities such as rate of force development (RFD) and power in developing sprinting speed [9]. Currently, there is very little long term, descriptive, observational research regarding the experience of elite 400 m sprinters who have followed combined resistance and running training programs. In terms of maximizing strength/power adaptations, block periodization and appropriate programming can result in superior strength/power gains [10,11,12,13,14]. Block periodization depends upon “stages”, each containing three fitness phases: accumulation, transmutation, and realization [15]. The sequenced order of these phases, along with appropriate programming, allows for early adaptations to further potentiate adaptations in the later blocks, termed phase potentiation [16,17,18]. For example, the development of work capacity and basic strength during the accumulation phase allows for greater development of maximal strength and power during the later phases of training [11,12,13,16,17,19,20,21]. The block model depends upon several levels of programming variation, including the use of heavy and light days, in which intensity and or volume may be increased or decreased through programmatic means such as sets, reps and relative intensities. This type of loading paradigm has the potential to enhance the recovery and adaptation processes, leading to a superior performance [11,12,13,14,22]. The combination of resistance training in a block periodized manner and track and field specific training resulted in a more efficient and efficacious improvement of maximal strength, rate of force development (RFD) and superior fatigue management in comparison to other forms of training. These studies [12,13] are characterized by a high degree of ecological validity and lend support to a combined approach in training, but more research is warranted within elite track and field settings including 400 m runners. Despite a growing evidence base for the value of a block periodized approach to training [15,16,17,18,23,24] there is a need for understanding whether/how elite athletes apply these strategies operate within the real-world annual training/competition calendar.

Several methods exist to better understand physical adaptations to a combined, periodized training plan including the isometric midthigh pull and vertical jump testing. The isometric midthigh pull (IMTP) is a commonly used method to monitor changes in performance potential through quantification of peak force (PF), force at a variety of epochs, and the RFD. The diagnostic ability of these measures may be of importance when considering time-constrained tasks within sports, such as jumping, sprinting, and change of direction [25,26]. Large negative correlations have been observed between PF, RFD and impulse (IP) and 0–5 m split time performance in highly trained sprinters [27]. Contrary, Healy et al. (2019) [28] found no statistically significant (but moderate to strong) relationships between IMTP peak force and relative peak force and sprint performance over 40 m with 10 m splits, among a group of twenty-eight national and international level sprinters. However, the authors did not measure 0–5 m or fully diagnose the force–time curve, examining measures of strength such as RFD or impulse. In regards to the maximal power capabilities and for identifying high-velocity power spectrum changes, the countermovement jump (CMJ) is primarily used. A substantial relationship between 400 m performance and average height of 30 s repeated CMJs was noted, but this relationship was not noted for a single CMJ height performance [4,29]. The lack of association between jump height (JH) and 400 m performance may be due to the fact that athletes may employ varying movement strategies (such as increasing the time of force application) to achieve a desired outcome (e.g., jump height) and therefore jump performance may be influenced by a variety of factors [30,31]. Undoubtedly the importance of a sprinter’s ability to produce high forces in a brief time period is paramount as elite sprinters have demonstrated foot contact times around 90 ms [6,7]. Comprehensive insight into athletes’ neuromuscular function can be gained through detailed analyses using force plates.

Particularly important is the taper/peaking strategy utilized towards the end of the macrocycle in an effort to allow the athlete to express their cumulative adaptations and increase the potential of success on the day of competition. Much of the conceptual framework of the fitness–fatigue paradigm and peaking for a specific competition deals with the alterations and fluctuations of an athlete’s preparedness across many blocks of training [32]. Force–time characteristics (underpins power expression) may be influenced by alterations in hormonal status which may be strongly affected by training variables: volume and intensity. Testosterone (T), cortisol (C) and the T/C ratio are often used as valuable tools for the evaluation of athlete preparedness [11,13,32,33]. The high-volume training typically observed in the accumulation block generally decreases T/C ratio as indicative of accumulated fatigue and training stress, whereas the decreased volume load observed in the transmutation and realization phases can result in pattern rebound and augments the T/C ratio, promoting preparedness [11,13,25,34]. This rebound effect has been associated with a greater ability to generate maximal forces, and explosive strength (rate of force development) [13,33,34,35,36]. Additionally, the T/C ratio may have an effect on the development of hypertrophy and tissue repair, which play a role in strength development. Increases in the size of a muscle from resistance training have been well established. However, little is known about the extent and time course of the changes in muscle hypertrophy as a result of resistance training combined with relatively high volume loads of specific 400 m training.

Despite the importance of monitoring physiological/performance adaptation and the growing popularity of using force plates [37] in monitoring strength/power capabilities in athletes, there are no (to our knowledge) studies related to 400 m sprinters. Therefore, understanding the magnitudes and direction of adaptations using case studies of elite level athletes can provide better insight into individual responses, serve as a better communication tool with coaches, and can also contribute to generating hypotheses for future research questions [38].

Thus, the purpose of this study was to examine the time course of the physiological and performance changes in an elite level 400 m male sprinter throughout four resistance training phases in combination with a sport-specific running program over a 16-week training period.

## 2. Methods

### Subject (Athlete)

The athlete was an elite level 400 m male sprinter (age 31 years, body mass: 74 kg, years of training: 15 and Personal Best (PB): 45.65 s. He was a 400 m Relay Indoor World Record holder from 2018 (3:01:77) and final participant of 4 × 400 m race in 2016 Olympics (Rio de Janeiro). Currently, he is part of the national team program preparing for the Tokyo Olympics. These data arose from the monthly monitoring program in which each athlete’s (from the National Team) physiological and motor abilities are routinely measured over the course of the season. The study was approved by the Institute of Sport Committee of Ethics, and written informed consent was obtained. The subject was informed of the benefits and risks of the investigation prior to signing an institutionally approved informed consent document to participate in the study. The study conformed to the recommendations of the Declaration of Helsinki.

## 3. Training Program and Testing Timeline

This study was a comparison of pre- and postblock testing results from four specific training phases throughout a single macrocycle leading up to a control indoor competition. The first testing session was held two weeks after the 2019 IAAF World Athletics Championships (after active recovery period). Testing dates corresponded to the start of a new block of training. The training program followed a single-factor block periodized design. The three periodization blocks consisted of four distinct training blocks. The initial training block (Accumulation 1: T1–T2) consisted of four weeks of high volume and low-to-moderate relative intensities, termed a Strength-Endurance Phase (SE). The second block of training (Accumulation II: T2–T3) consisted of four weeks of moderate volumes at higher intensities, termed a Maximal Strength Phase. The third block (Transmutation) termed Strength–Speed consisted of 4 weeks of low volumes, and high intensities combined with more velocity dominant exercises. The emphasis within this phase of training is to move relatively heavy loads quickly to enhance RFD characteristics [17,39]. The final block (Realization) of training consisted of 4 weeks of complex training where the primary exercises were combined with plyometric-type exercises which place greater emphasis on the high velocity end of the force-velocity spectrum while maintaining strength qualities (Speed–Strength phase). Each training session was completed within 1.5 h. The basic structure of the block periodized training program is presented in Table 1. Testing occurred at the beginning of the week 1 (T1), and after completion of week 4 (T2), 8 (T3), 12 (T4), 16 (T5).

Following baseline testing, the athlete completed both resistance (RT) and running programs on alternating days. RT was completed on Mondays, Wednesdays, and Fridays, whereas a rudimentary running program was completed on Tuesdays, Thursdays and Saturdays. At the onset of T2, RT frequency was reduced to 2 day/week while running frequency was increased to 4 day/week. RT was completed on Tuesdays and Thursdays while running training was completed on Mondays, Wednesdays, Fridays and Saturdays. Resistance training loads were prescribed using relative intensities for a given set and repetition range [14,17,19]. This approach has been shown to produce superior performance adaptations when compared to traditional loading methods such as repetition maximum zones [14]. Exercises employed in each block are presented in Table 2.

Running training intensity was based on a distribution of training into 7 specific intensities zones presented in Table 3. It should be added that this running training method has been used since 1994 when the head coach became the Polish national 400 m relay male team’s coach. This method helped the Polish 400 m relay team reach the world record for the fifth time in history (2:58:00 Uniondale, New York, Goodwill Games) and achieve the Indoor World Record in 2018. The average lactate and RPE values in each training mean are presented based on more than 40 years of collecting data on national team athletes. Due to the fact that test dates are dictated by resistance training programs, it is reasonable to present running training programs with respect to this. Although this study is primarily concerned with adaptations to resistance training, physical performance potential is affected by external stressors including physical stress from both resistance training and running training. Therefore, it is important to analyze all aspects of the athlete’s physical preparation before testing to better understand their response to training. Therefore, the frequency of each type of training (units·week^−1^) and percentage distribution to each resistance training block was recorded (Table 4).

## 4. Blood Collection

All testing sessions were completed at the Polish Institute of Sport. On each occasion, the subject arrived at the Institute 24 h before testing began. Blood draws were conducted by a medical doctor at the same time of day (7:00 to 9:00 a.m.) to account for diurnal variation [41], but at least 45 min after waking to eliminate early morning variation in hormones [42,43]. Commercially available ELISA kits (DRG Diagnostics, Marburg, Germany) were used to determine total cortisol and testosterone concentrations in serum. All samples were assayed in duplicates and the coefficients of variation of the intra-assays were less than 6% for cortisol and testosterone; moreover, the reference material (Bio Rad Laboratories, Plano, TX, USA) was attached to each analytical run. The hormonal analyses were carried out in the laboratory of the Department of Biochemistry with an implemented quality system (with accreditation of the Polish Centre for Accreditation no. AB946).

## 5. Body Composition

Body composition was assessed via DXA (Hologic Inc., Bedford, MA, USA; Apex Software Version 3.3). Athletes were asked to arrive fasted, but hydrated, and rested to their testing session wearing only lightweight athletic clothing. Upon arrival, athletes were asked to remove all metal to avoid interference with the DXA scan. Height, weight, ethnicity, sex, and date of birth were entered into the DXA computer. Athletes were asked to lie supine in the middle of the scanning table with all extremities fitting inside of the measuring parameter. If an athlete was taller than the parameter of the scan, they were positioned so that their head was as close to the top of the scan parameter as possible and the ends of their toes were excluded from the scan. DXA test-retest reliability for male from Kutáč et al. (2019) [44]: intraclass correlation coefficient (ICC) = 0.99 and typical error of measurement (TEM) = 0.29 kg for body mass (BM), ICC = 0.98 and TEM = 0.52 for fat mass in kg (FM), ICC = 0.98 and TEM = 0.66% for % fat mass (FM %), ICC = 0.99 and TEM = 0.42 kg for fat–free mass and ICC = 0.99 and TEM = 0.02 kg for bone mineral content.

## 6. Vertical Jump Assessments

Countermovement jumps (CMJ) were assessed at 5 time points as indicated in Table 1. Following a standardized dynamic warm-up, subject performed 2 warm-up CMJs with their hands-on-hips and feet shoulder-width apart and self-selected countermovement depth. Following 50% and 75% effort warm-up jumps, 3 maximal effort CMJs were performed on a force plate (“JBA” Zb. Staniak, Poland) with a 400 Hz sampling rate. The force plate was connected via an analog-to-digital converter to a PC with the MVJ v.3.4 software (“JBA” Zb. Staniak, Poland). The vertical component of ground reaction force was used to calculate a number of kinetic and kinematic variables characterizing each jump. Kinetic and kinematic data were collected and processed using MVJ v.3.4 software (“JBA” Zb. Staniak, Poland). Height of the rise of the body center of mass (COM) during vertical jumps was calculated from the recorded ground reaction force of the platform [45]. The onset of profile calculation for each jump considered to have occurred 10 milliseconds prior to the instant when vertical force had changed to 2% in relation to the body weight as derived during the silent period of standing. The jump analysis application calculates net impulse via trapezoidal integration of the force–time data, while center of mass (COM) velocity is calculated by normalizing the impulse to body mass. Jump height is then determined from the impulse-momentum theorem using takeoff velocity via TOV jump height = TOV^2^/2 g where TOV = takeoff velocity and g = 9.81 m/s^2^ [46]. Aside from the flight phase, three additional phases are determined prior to takeoff: unweighting, braking (eccentric), and propulsion (concentric). The unweighting phase represents the area of the force–time curve that is below BW and instants of peak negative COM velocity. The braking phase continues from the end of the unweighting phase until the instant COM velocity increases to zero. The propulsion phase of the CMJ was deemed to have occurred between the instant that COM velocity equal or exceeded 0 and the instant of takeoff. Readers are referred to McMahon et al. (2018a) [37] for further discussion on phase determination. Eccentric and concentric force and power (peak) were defined as the maximum or average vertical force and power values, respectively, attained during the eccentric and concentric phases of the jump. The ratio between average eccentric (ECC) power and average concentric (CON) power was also determined and termed the power ratio [47]. Reactive strength index modified was calculated as jump height divided by movement time [48]. Previous data from our lab indicate high reliability (ICCs 0.81–0.93) and low variability (CV < 10%) for all CMJ variables. We demonstrated high reliability (ICCs range = 0.81–0.93) and acceptable variability (CV < 10%) for all CMJ variables in our lab.

## 7. Isometric Midthigh Pull Assessments

Isometric peak force (IPF) and accompanying RFD were assessed from isometric midthigh pulls (IMTP) performed at each testing time point. Specifically, RFDs from 0 to 100 ms (RFD 100), from 0 to 200 ms (RFD 200), and from 0 to 300 ms (RFD 300) were considered. During 400 m races, ground contact times of between 110 ms and 145 ms have been reported [49,50,51]. Following a standardized warm-up, the athlete was positioned in a custom-built power rack with an affixed bar. The athlete’s internal knee and hip angles were measured manually using a goniometer and were required to be 130° and 150°, respectively. The distance between the force plate top surface and the weightlifting bar was set in with an accuracy of 2 mm. Each power rack contained force plate (“JBA” Zb. Staniak, Poland) with a 400 Hz sampling rate which has been previously shown to be reliable and valid [52,53]. The athlete was secured to the bar using straps and athletic tape to eliminate grip strength as a confounding variable during testing. Prior to maximal effort trials, a 50% and a 75% effort warm-up pull was completed, separated by 60 s of rest. Three minutes of rest was given following the final warm-up effort. The athlete completed 3 maximal effort IMTP trials and was instructed to “pull as fast and as hard” as he could. The athlete continued to pull until peak force dropped off. Additional trials were completed if the IPF differed between trials >250 N or if there was a >200 N countermovement in any trial. Verbal encouragement was provided during every IMTP effort. The pretension level was standardized every time by visual feedback of the force–time curve. Zero moment of time was assumed when the limit value of force 300 N was obtained. In the case where a value of 300 N occurred between samples resulting from the sampling rate (400 Hz) the zero time was determined by taking a linear course of force between samples. In the same way, the force values were determined at specific RFD time moments. The course of isometric effort force (IMTP) was digitally smoothed with a 20 Hz four-pole low-pass Butterworth filter. Kinetic data were processed using MTP v. software (“JBA” Zb. Staniak, Poland). IMTP pretension was not standardized during the first testing session. Because pretension has an effect on RFD, RFD data from the first testing session cannot be directly compared with the following testing sessions and has been excluded from analysis. Peak force was not significantly affected by pretension levels and is reported from the first testing session. All kinetic data were also divided by body mass to allow for a normalized comparison of these data between time points. Reliability data of all IMTP variables for our lab are listed in Table 5.

## 8. Statistical Analyses

Descriptive statistics are reported as mean ± SD. Comparisons between baseline (T1) and testing data points (T2, T3, T4 and T5) throughout the 16-week training mesocycle were assessed through the percentage (%) difference in change scores. (ES) data were calculated to determine the magnitude of the change score and were assessed using the following criteria for highly trained subjects [54] <0.25 = trivial, 0.25–0.50 = small, 0.5–1.0 = moderate, 1.0–2.0 = large, and >2.0 = very large.

## 9. Results

### Testosterone: Cortisol Ratio, Testosterone, and Cortisol

The mean values for the testosterone, cortisol and T/C ratio are shown in Table 6.

The T/C ratio changes was affected mainly by greater fluctuations in cortisol than testosterone. The greatest percent change in cortisol upward trend was noted from T1 to T2 which coincides with the largest downward trend of T/C ratio (37% decrease). The lowest value of T/C ratio was observed in T4; however, in this case, it was primarily due to the decrease in testosterone (−19%) concentration while cortisol concentration was relatively stable from T2–T4. The largest upward trend of T/C ratio was noted from T4 to T5 (86%) which was the effect of 32% decrease and 26% increase of cortisol and testosterone, respectively.

## 10. Body Composition

Minor changes (<2%) were observed in body mass at all five testing points during the study (Table 7.) The greatest changes were observed between T1–T4 (12th week) in fat mass (−3%) and fat–free mass (+3.7%) alterations. An abrupt decrease in body mass was observed between T4–T5, which also corresponded to a decrease in fat free mass and an increase in percent fat and fat mass. The previous literature has suggested these changes in body mass may be a function of a competitive taper [55,56].

## 11. Vertical Jumping

For each gross measure, the mean output of the three CMJs trials was used for further analysis. Alterations in CMJs phase variables between testing dates are presented in Table 8. Results are expressed as mean ± SD.

Effect sizes and % changes between testing time points are presented in Table 9. During the SE phase (T1–T2) there were small to large decreases in the number of CMJ performance. Jump height, take-off velocity and concentric impulse showed the greatest decrements. From T2 to T3, after the maximum strength phase, there were only trivial to small alterations in most variables. Large increases in jump height, take-off velocity, peak negative velocity, CON and ECC impulse, power ratio and CMJ depth (greater dip) between T3 and T4 were observed. Small increases in contraction time were noted, mostly due to moderate increase in propulsion time rather than changes in unweighting or braking phase duration. After the speed-strength phase (T4–T5) the large increases in JH, take-off velocity, RSImod, Peak and mean CON power and moderate increases in peak force (CON and ECC) and ECC mean power. A moderate decrease in contraction time was found, mostly due to moderate and small decreases in unweighting and braking phase time, respectively, as there was no change in propulsion phase time. When comparing T5 to pretraining cycle values (T1) there was a large increase in JH, CON and ECC Peak Power, take-off and peak negative velocity, CON and ECC impulse and in power ratio.

## 12. Isometric Midthigh Pull

**Figure 1:**
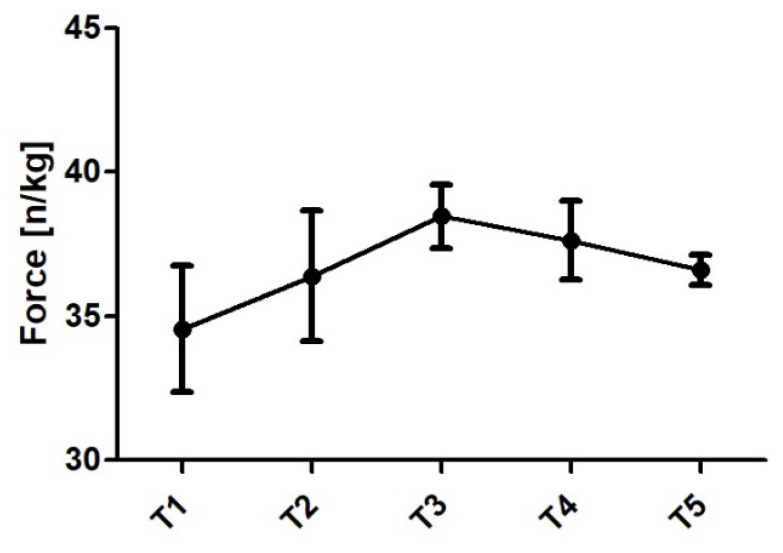
Peak force from the IMTP test.

**Figure 2:**
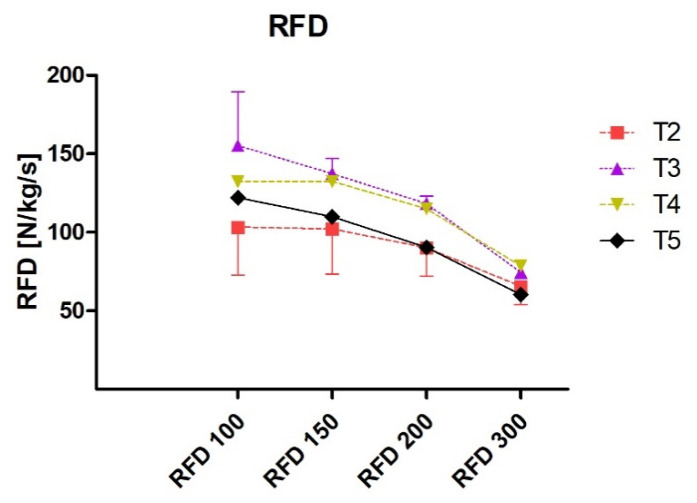
Rate of force development in 100, 150, 200 and 300 ms throughout 16-week training.

Relative peak force (N/kg) alterations are presented in Figure 1 and raw values in Table 10. The upward and downward phase of peak force changes coincides with alterations in fat-free mass (reverse U shape). From T1 to T2 the subject displayed moderate (ES = 0.88; Δ% = 8.6%) increase in relative peak force and this upward trend was observed until T3 (week 8). From T2 to T3 (ES = 0.63; Δ% = 5.74%) increase was noted. A small decrease from T3 to T4 (ES = −0.26; Δ% = −2.27%) and from T4 to T5 (ES = −0.3; Δ% = −2.63%) was reported. There was large increase in RFD 100 (ES = 1.5 Δ% = 50.43%), RFD 150 (ES = 1.51; Δ% = 34.43%), RFD 200 (1.76, Δ% 31.4%), and moderate increase in RFD 300 (ES = 0.84; 13.46%) from T2 to T3 (Figure 2). RFD 100 trended downward from T3 to T4 (ES = −0.66; Δ% = −14.87%) while no substantial (trivial) changes were observed for 150 and 200 ms time epochs and small upward trend (ES = 0.38; Δ% = 5.46%) was noticed for RFD 300. From T4 to T5 where small downward change was observed for RFD 100 (ES = −0.29; Δ% = −7.6) while moderate to large decrease in RFD 150 (ES = −0.97; Δ% = −16.9), 200 (−1.53; Δ% = −21.2), and 300 (−1.74; Δ% = −23.3%) was reported. Raw RFD values are presented in Table 11.

## 13. Discussion

The results of the current study support the use of a block periodized RT program for improving relative strength and power qualities in an elite 400 m runner. The basis of block periodization can be traced to the work of Verkoshansky and Issurin. Verkoshansky described the use of programmed concentrated loads, and a long-term lag of training effects between the initiation of a training stimulus and when the effects are realized [57]. Issurin described how appropriate programming can produce residual effects which can potentiate adaptations in a subsequent phase [15,23]. These ideas have later been termed phase potentiation programming [16]. The results of the current study support the use of a block periodized and a phase potentiation program with alterations in body composition during the first phase of training allowing for future gains in maximum strength and ultimately power during the later phases.

There was an overall increase in relative strength from T1–T5 of 6.04% (ES = 0.81). Relative strength peaked following the maximum strength training block with an 8.6% increase from T1–T2 and 5.7% increase from T2–T3. However, once the concentrated load transitioned to power development there was a small decrease in relative strength from T3–T4 (−2.27%) and T4–T5 (−2.63%). Despite these reductions, relative strength remained elevated above baseline. These increments in relative strength may have contributed to the improvements in dynamic jump performance. Suchomel et al. (2016) [58] indicate there may be a relative strength threshold above which greater gains in power are possible. This idea is supported by the observations of Wetmore et al. (2020) [21] indicating that stronger subjects realized greater gains in power-related measures during a realization block. Our current results support this theory as peak eccentric and concentric power, mean eccentric and concentric power as well as power ratio all improved in the final two blocks following an increase in relative strength.

Jump height increased 5.7% from T1–T5, which corresponded to a large effect size. The concentric impulse (9.4%), eccentric impulse (3.4%) and power ratio (18.7%) all substantially increased over the course of the training program. The trends of changes in jump variables from block to block were reflective of the concentrated load for each individual block. For example, as expected, the jump height, velocity at takeoff and power variables all decreased following the first two blocks of training. This period included the highest volumes of training and therefore greater fatigue. However, when the concentrated loading switched towards developing maximum strength and power, jump variables supercompensated above baseline values. These results are quite meaningful as previous work has demonstrated the strong relationship (r^2^ = 0.81) between jump height and sprinting performance in elite sprinters [59].

Body composition changes as well as hormonal balance changes may also help explain the physical adaptations to the training program. For example, one goal of early training is to alter body composition, which can be achieved through higher volume training. This may also cause an increase in overall stress, which may reduce T:C. In the current study, fat mass was reduced by 2.3% and lean mass increased by 1.4 kg from T1–T3. Additionally, T:C was reduced by 37% from T1–T2. However, during the later phases of training, fatigue management is paramount in order to allow for other qualities such as power to be expressed. During the last phase of training, body mass was substantially reduced by 2.2 kg but T:C increased by 86%. The balance of these two factors may have affected the peak in performance in vertical jump variables and the athlete’s ability to run 400 m.

Lastly, our results support the theoretical basis of a competitive taper. Previous research [60] has indicated that the purpose of a competitive taper is to lower fatigue while increasing readiness. This can be accomplished by reducing training volume, intensity, and possibly training cessation. The current literature recommends a taper of 7–14 days (REFS). Expected outcomes of a taper may include increased power outputs, greater T:C ratios, increased strength:body mass ratios and ultimately better performance [61,62]. In our current study, the resistance training volume as well as the intensity were reduced for 7 days prior to the final testing session. As mentioned previously, our results showed an 86% (see above) increase in T:C as well as increases in dynamic performance variables. Additionally, our subject had a reduced body mass from T4–T5, perhaps as a result of reduced training volume and could possibly have contributed to the greater jump power observed in T5.

## 14. Conclusions

In conclusion, across the macrocycle the athlete appears to have increased preparedness and responded favorably to the planned training. A manner generally “fitting” of the phase-based progression of higher volume to lower volume along with a gradual rise in training intensity and sport specific preparation. This is based primarily on the alterations in T and T:C ratio as well the neuromuscular alterations across the phases. During the higher volume training the athlete demonstrated lower T and T:C and lower neuromuscular readiness (based on the jump data). Particularly noteworthy is the elevated preparedness at the of the taper (e.g., highest T and best jump performance). In high level athletes an important factor is not simply “does an athlete improve” but the meticulously controlling of when (e.g., a major competition).

While case studies, particularly case studies performed on very advanced athletes in a highly ecologically valid setting can be quite valuable, studies such as this are not without limitations. The nature of a case study limits external validity and the ability to generalize data to a population. It is important for coaches to understand and appreciate the advanced level of the sprinter observed and that elite training and coaching is very nuanced and based on the individual. Second, as mentioned in the methods, the track running program was designed by the national team coach and thus could not be directly manipulated along with the RT program. The RT program was manipulated to “fit” the running program, so as to not produce adverse effects (i.e., poor fatigue management), subject, case studies, involving detailed reporting of the athletes’ training over extended periods of time can provide important insight into high level sport. This study, along with many others of similar design, suggests the importance of: (1) focused training periods, (2) heavy and light training days, (3) and detailed, inclusive planning [63,64,65].

## Figures and Tables

**Table 1:** Training program structure.

Block	Week	Sets	Repetitions	Intensity/Day					
				Monday	Tuesday	Wednesday	Thursday	Friday	Saturday
Strength Endurance	1	3	10 *	M	Running	ML	Running	L	Running
	2	3	10 *	MH	Running	M	Running	L	Running
	3	3	10 *	H	Running	MH	Running	ML	Running
	4	3	5 *	M	Running	M	Running	L	Running
Max Strength Phase	5	3	5 *	H	Running	MH	Running	M	Running
	6	3	5 *	H	Running	H	Running	M	Running
	7	3	5 *	VH	Running	H	Running	MH	Running
	8	3	5 *	ML	Running	L	Running	L	Running
Strength–Speed	9	3	3 *	Running	H	Running	MH	Running	Running
	10	3	3 *	Running	VH	Running	H	Running	Running
	11	3	3 *	Running	VVH	Running	M	Running	Running
	12	3	3 *	Running	ML	Running	L	Running	Running
Speed–Strength	13	3	3 *	Running	MH	Running	L	Running	Running
	14	3	3 *	Running	H	Running	M	Running	Running
	15	3	3 *	Running	VH	Running	MH	Running	Running
	16	3	3 *	Running	L	Running	L	Running	Running

Note: SE = Strength—Endurance, SP = Strength—Power, VL = very light (65–70%), L = light (70– 75%), ML = medium light (75–80%), M = medium (80–85%), MH = medium heavy (85–90%), H = heavy (90–95%), VH = very heavy (95–100%). Intensities are based off a set-rep best system [14,40]. * represent a single drop set at approximately 60% of the working sets.

**Table 2:** Resistance training program and organization.

Day	Strength—Endurance	Strength	Strength–Speed	Speed–Strength
Monday	Back squat Overhead press Lunges Bench press	Back squat Overhead press Dumbell step-up Bench press		
Tuesday			¼ back squats and squat jumps Push press and vertical medicine ball toss Midthigh pull from box and CG CM shrug Nordics * Bench press	Depth jumps * Hurdle hops Loaded countermovement jump (CMJ) Back Squat and box jumps ($)
Wednesday	Clean pull to knee Pull-up Stiff leg deadlift Band assisted Nordics	Clean pull from floor Clean pull from knee Pull-up Stiff leg deadlift		
Thursday			CG CM shrug Push press and vertical medicine ball toss Half squat and AEL (#) Deadlift Barbell prone row	Assisted band jumps AEL (#) ¼ loaded CMJ Clean pull and power clean ($)
Friday	front squat overhead press lunges bench press	front squat midthigh pull bench press stiff leg deadlift		

* Depth jumps from 30 cm; $—complex training. Back squat at VVH—4 min rest–box jumps. Clean pull at VVH—4 min rest–power clean, #—accentuated eccentric loaded jumps with 2 × 15 kg dumbbells in eccentric phase. CG = clean grip, CM = countermovement, AEL = accentuated eccentric loading.

**Table 3:** 400 m training means.

	Training Zone/Kind of Training	Lactate (mmol/L)	Rpe	Description	Training Example
1	Aerobic long intervals	<4 mmol	<3	8–10 km interval run with changing intensity	4 km jog 4× (2′/2′) 4× (1:30″/1:30″)
2	Speed endurance type 1	6–8 mmol/L	3–5	100–200 m runs with medium and long intervals and submaximal pace (70–85% Vmax)	4× (5 × 100 m) 1st set: 5 × 100 (16 s)/1:15″ 2nd set: 5 × 100 (15 s)/1:30″ 3rd set: 5 × 100 (14 s)/1:45″ 4th set: 5 × 10 (13 s) Interset recovery: 4′, 6′ and 8′ after 1st, 2nd and 3rd set respectively
3	Speed endurance type 2	10–12 mmol	5–7	100–200 m runs with medium and long intervals and submaximal to maximal pace (85–100% Vmax)	5× (2 × 200 m) in 32″; 30″; 28″; 26″ (spikes); 24″ (spikes Inter-repetitions recovery 2′, 2′, 2′, 3′, 3′ Interset recovery: 8′, 10′, 12′, 14′
4	Special endurance	10–15 mmol	7–9	200–400 m runs with medium and long intervals with submaximal to near maximum pace (70–95% Vmax)	7 × 400 m in 80″; 76″; 72″; 68″; 64″; 60″; 56″ Rest between reps 2′, 4′, 6′, 8′, 10′ 12′
5	Tempo endurance	12–17 mmol	8–10	300–500 m runs with medium interval and submaximal intensity	4 × (500 m + 300 m) 1st set 500 m in 1′40″ 300 m in 57″ (5′ rest between) 4th set 500 m in 1′10″ 300 m in 39″ (10′ rest between sets)
6	speed	No data	4–5	40–120 m runs with near maximal intensity (95–100% Vmax)	2 × 40 m 2 × 60 m 2 × 80 m 1 × 120 m Inter repetitions recovery: 4 min Interset recovery 8 min
7	running strength	No data	4–5	Bounding, skipping and explosive throws	Jumps to 30 m or 10 repetitions Standing long jumps 3–10 alternate leg hops or bounding

**Table 4:** Distribution and number of running sessions in each block throughout 16 weeks.

Block	Aerobic Intervals	Speed Endurance Type 1	Speed Endurance Type 2	Special Endurance	Tempo Endurance	Speed	Running Strength	Number of All Training Sessions
Strength endurance	50%	25%	25%	0%	0%	0%	0%	24
Strength	0%	25%	33%	25%	16%	16%	0%	24
Strength–speed	5%	11%	16%	16%	16%	5%	27%	40
Speed–strength	5%	5%	35%	50%	0	5%	0	5

**Table 5:** Reliability of IMTP variables.

Variable	ICC	CV (%)
PF	0.97	4%
RFD100	0.85	12%
RFD150	0.91	10%
RFD200	0.93	7.7%
RFD300	0.94	6.4%

**Table 6:** Anabolic-to-catabolic hormone alterations over the 16 weeks.

	Descriptive Values					Percent Change (Δ%) for T, C and T/C			
	T1	T2	T3	T4	T5	T2–T1 Δ%	T3–T2 Δ%	T4–T3 Δ%	T5–T4 Δ%
Testosterone [nmol/L]	27	28.9	30.9	25	31.5	7%	6.90%	−19.10%	26%
Cortisol [nmol/L]	305	518	557	573	386	69%	7.50%	2.87%	−32%
T/C [nmol/L]	8.9	5.6	5.5	4.4	8.2	−37%	−1.78	−20%	86%

**Table 7:** Body Composition Alterations across the 16 weeks.

	T1	T2	T3	T4	T5
Body mass	76.2	75.1	75.8	76.3	74.1
BMI [kg/m^2^]	22	21.6	22	22	21.6
Fat mass [kg]	8.8	7.9	7.0	6.5	6.9
Fat mass [%]	11.5	10.6	9.2	8.5	9.3
Fat–free body mass [kg]	64.3	63.9	65.7	66.7	64.6
Bone mineral content [kg]	3.2	3.2	3.1	3.2	3.2

**Table 8:** Countermovement jump kinetic and kinematic data across 16 weeks.

	T1	T2	T3	T4	T5
Jump Height [cm]	53 0.01	50 0.00	49.8 0.01	52.3 0.00	55.9 0.00
RSI mod	0.815 0.04	0.759 0.05	0.748 0.00	0.771 0.02	0.852 0.01
Velocity at take–off [m/s]	3.21 0.03	3.12 0.04	3.11 0.05	3.18 0.01	3.3 0.02
Time to take off [s]	0.65 0.01	0.66 0.04	0.66 0.02	0.67 0.02	0.65 0.01
Unweighting phase time [s]	0.295 0.006	0.310 0.035	0.314 0.006	0.316 0.025	0.299 0.015
Braking phase time [s]	0.141 0.009	0.141 0.009	0.142 0.014	0.143 0.006	0.138 0.005
Propulsion phase time [s]	0.215 0.005	0.211 0.001	0.210 0.014	0.219 0.004	0.219 0.006
CMJ depth [cm]	−38 0.00	−36.2 0.00	−35.6 0.02	−38.3 0.01	−39.7 0.01
Peak negative velocity [m/s]	−1.53 0.05	−1.48 0.03	−1.49 0.03	−1.64 −0.09	−1.68 0.03
Peak Eccentric Force [N·kg^−1^]	17.3 1.04	16.43 0.8	16.43 0.97	16.6 0.81	17.4 0.2
Peak Concentric Force [N·kg^−1^]	17.6 0.95	17.23 0.49	16.96 1.06	17.16 0.3	17.9 0.36
Peak Eccentric Power [W·kg^−1^]	12.4 2.49	11.6 1.3	11.83 0.92	14.16 2.51	15.53 0.66
Peak Concentric Power [W·kg^−1^]	41.7 1.13	40.36 1.44	41.23 1.04	41.06 0.92	44.6 1.68
Mean ECC Power [W·kg^−1^]	8.04 1.17	7.49 0.89	7.62 0.50	9.1 1.45	9.85 0.26
Mean CON Power [W·kg^−1^]	24.56 1.07	23.53 0.56	23.56 0.86	23.66 0.20	25.46 0.86
Power Ratio [%]	32.64 3.25	31.81 3.39	32.31 1.11	38.52 6.44	38.7 2.27
Eccentric Impulse [N·s^−1^·kg^−1^]	1.49 0.06	1.43 0.02	1.46 0.04	1.6 0.1	1.63 0.03
Concentric Impulse [N·s^−1^·kg^−1^]	3.28 0.04	3.20 0.04	3.18 0.04	3.25 0.01	3.39 0.01

**Table 9:** Effect sizes and % change from CMJ test.

	T1 vs. T2	T2 vs. T3	T3 vs. T4	T4 vs. T5	T1 vs. T5
	ΔES; Δ %				
Jump height [cm]	−1.58 −5.7%	−0.16 −0.6%	1.34 4.0%	2.0 7.7%	1.60 5.7%
RSImod	−1.04 −7.3%	−0.33 −1.3%	0.46 2.7%	1.63 10.4%	0.72 3.7%
Velocity at take–off [m/s]	−1.61 −2.8%	−0.18 −0.3%	1.37 2.6%	2.08 3.4%	1.67 2.8%
Time to take off [s]	0.26 1.5%	0.09 0.6%	0.31 2%	−0.54 −3.2%	0.13 0.8%
Unweighting phase time [s]	0.43 4.85%	0.12 1.29%	0.07 0.74%	−0.5 −5.27%	0.12 1.35%
Braking phase time [s]	0 0%	0.06 0.7%	0.06 0.7%	−0.34 −3.5%	−0.2 −2.1%
Propulsion phase time [s]	−0.31 −1.9%	−0.11 −0.5%	0.8 4.3%	0 0%	0.37 1.9%
CMJ Depth [cm]	−0.78 −4.5%	−0.26 −1.7%	1.18 7.3%	0.62 3.7%	0.75 4.5%
Peak negative velocity [m/s]	−0.58 −3.3%	0.18 1.4%	1.55 9.3%	0.47 2.4%	1.62 9.8%
Peak eccentric force [N·kg^−1^]	−0.67 −5.0%	0.0 0.0%	0.13 1.0%	0.61 4.8%	0.08 0.6%
Peak concentric force [N·kg^−1^]	−0.33 −2.3%	−0.24 −1.2%	0.18 1.2%	0.66 4.1%	0.27 1.7%
Peak eccentric power [W·kg^−1^]	− 0.29 −6.5%	0.08 2.0%	0.84 19.8%	0.49 9.6%	1.12 25.2%
Peak concentric power [W·kg^−1^]	−0.66 −3.1%	0.43 2.0%	−0.08 −0.2%	1.75 8.5%	1.44 7.0%
Peak eccentric power [W·kg^−1^]	−0.29 −6.5%	0.08 2.0%	0.84 19.8%	0.49 9.6%	1.12 25.2%
Mean ECC power [W·kg^−1^]	−0.36 −6.84%	0.08 1.73%	0.98 19.51	0.49 8.23%	1.19 22.5%
Mean CON power [W·kg^−1^]	−0.84 −4.2%	0.02 0.14%	0.08 0.42%	1.46 7.6%	0.73 3.66%
Power ratio [%]	−0.14 −2.5%	0.08 1.6%	1.05 19.2%	0.03 0.6%	1.03 18.7%
Eccentric impulse [N·s^−1^·kg^−1^]	−0.61 −4%	0.25 2.1%	1.47 9.6%	0.27 1.9%	1.38 9.4%
Concentric impulse [N·s^−1^·kg^−1^]	−1.4 −2.4%	−0.41 −0.6%	1.31 2.2%	2.44 4.3%	1.9 3.4%

**Table 10:** Relative peak force data [N·kg^−1^]. Mean (±SD).

Peak Force [N·kg^−1^]	T1	T2	T3	T4	T5
	34.51	36.40	38.49	37.62	36.63
	(2.17)	(2.24)	(1.09)	(1.35)	(0.51)

**Table 11:** Relative RFD data [N·kg^−1^·s^−1^] Mean (±SD).

RFD	T2	T3	T4	T5
RFD 100	103.23 (30.45)	155.30 (34.14)	132.20 (16.21)	122.07 (6.76)
RFD 150	102.03 (28.72)	137.17 (9.70)	132.40 (8.07)	109.90 (9.22)
RFD 200	90.00 (17.87)	118.27 (4.70)	114.93 (8.12)	90.47 (10.14)
RFD 300	65.6 (11.6)	74.4 (2.3)	78.5 (5.7)	60.2 (6.9)

## Data Availability

The data that support the findings of this study are available from the corresponding author upon reasonable request.
